# Short-course radiotherapy combined with CAPOX and Toripalimab for the total neoadjuvant therapy of locally advanced rectal cancer: a randomized, prospective, multicentre, double-arm, phase II trial (TORCH)

**DOI:** 10.1186/s12885-022-09348-z

**Published:** 2022-03-15

**Authors:** Yaqi Wang, Lijun Shen, Juefeng Wan, Hui Zhang, Ruiyan Wu, Jingwen Wang, Yan Wang, Ye Xu, Sanjun Cai, Zhen Zhang, Fan Xia

**Affiliations:** 1 Department of Radiation Oncology, Fudan University Shanghai Cancer Center, Shanghai, China; 2 Department of Oncology, Shanghai Medical College, Fudan University, Shanghai, China; 3 Shanghai Key Laboratory of Radiation Oncology, Shanghai, China; 4 Department of Colorectal Surgery, Fudan University Shanghai Cancer Center, Shanghai, China

**Keywords:** Locally advanced rectal cancer, Immunotherapy, Short-course radiotherapy, Total neoadjuvant therapy, Complete response

## Abstract

**Background:**

For patients with locally advanced (T3-4/N +) rectal cancer (LARC), the standard treatment is neoadjuvant chemoradiotherapy combined with total mesorectal resection, which greatly decreases local recurrence but does not improve overall survival. For patients who achieve a complete clinical response (cCR) after nCRT, a “Watch & Wait” (W&W) approach can be received to improve quality of life. Currently, total neoadjuvant therapy (TNT) has been demonstrated to increase the complete response rate and achieve early control of distant metastasis. Recent studies have shown promising synergistic effects of the combination of immunotherapy (PD-1/PD-L1 antibodies) and radiotherapy. Thus, for LARC patients, the combination of immunotherapy and TNT is likely to further improve the rate of complete response and prognosis. The disparities between induction therapy and consolidation therapy need to be investigated.

**Methods:**

TORCH is a randomized, prospective, multicentre, double-arm, phase II trial of short-course radiotherapy (SCRT) combined with chemotherapy and immunotherapy in LARC. 130 LARC patients will be treated with the TNT approach and assigned to the consolidation arm and induction arm. The consolidation arm will receive SCRT, followed by 6 cycles of capecitabine plus oxaliplatin (CAPOX) and Toripalimab. The induction arm will first receive 2 cycles of CAPOX and Toripalimab, then receive SCRT, followed by 4 cycles of CAPOX and Toripalimab. Both groups will receive curative surgery or the W&W strategy. The primary endpoint is the complete response rate (rate of pCR plus cCR). The secondary endpoints include the grade 3–4 acute adverse effects rate, 3-year disease-free survival (DFS) rate, 3-year local recurrence-free survival (LRFS) rate, 3-year OS rate, rate of surgical complications and quality of life (QoL) scores. The “pick the winner” method is used to investigate the better treatment regimen. The trial was opened on 13th April 2021, and the first patient was recruited on 6th May 2021.

**Discussion:**

TORCH will investigate whether SCRT combined with chemotherapy and Toripalimab can achieve better complete response rates, good tolerance and prognosis in LARC patients. This is the first clinical trial to compare the efficacy of induced immunotherapy and consolidative immunotherapy based on the TNT strategy.

**Trial registration:**

Trial Registration Number and Date of Registration: ClinicalTrials.gov NCT04518280, August 15, 2020.

## Background

For patients with locally advanced (T3-4/N +) rectal cancer (LARC), the standard treatment is neoadjuvant chemoradiotherapy (nCRT) combined with total mesorectal resection (TME), including long-course chemoradiotherapy (LCRT, 50 Gy/25 Fx, concurrent fluorouracil (5-FU) or capecitabine) and short-course radiotherapy (SCRT, 25 Gy/5 Fx). nCRT significantly reduced the tumour stage, increased the sphincter preservation rate, and resulted in a very low rate of local recurrence (approximately 6–7%). However, overall survival (OS) did not benefit, and distant metastasis became the main cause of treatment failure [1]. Patients with pCR after surgery were verified to have a better prognosis [2]. Meanwhile, for patients who achieve a complete clinical response (cCR) after nCRT, a “Watch & Wait” (W&W) approach can be adopted to preserve the organ and greatly improve quality of life [3, 4]. It has been demonstrated that the W&W approach has a prognosis similar to that of patients who receive radical surgery [5].

To increase the degree of tumour regression and pCR/cCR rates, improve compliance with perioperative chemotherapy and achieve early control of distant metastasis, researchers have tried to move adjuvant chemotherapy ahead of surgery (consolidation chemotherapy) or ahead of nCRT (induction chemotherapy) and even to perform total neoadjuvant therapy (TNT). This treatment model was already demonstrated to achieve a higher rate of pCR [6, 7]. The PRODIGE 23 trial [8] showed that 6 cycles of FOLFINOX before nCRT could lead to higher pCR (27.5% vs. 11.7%), 3-year DFS% (75.7% vs. 68.5%) and 3-y DMFS (78.8% vs. 71.7%) compared with the standard LCRT. The OPRA trial [9] recruited lower LARC patients with little possibility of preserving the anus at baseline, performed the TNT approach and compared induction treatment and consolidation treatment. The results showed that the two TNT arms both obtained an approximately 50% organ preservation rate, and the 3-year DFS% was similar to previous data. Compared with LCRT, SCRT combined with consolidation chemotherapy can still achieve good outcomes. The RAPIDO trial [10] showed that SCRT followed by 6 cycles of CAPOX or 9 cycles of FOLOFX (TNT approach) for high-risk LARC can significantly reduce the rate of disease-related treatment failure (DrTF, 23.7% vs. 30.4%), reduce the rate of distant metastasis (19.8% vs. 26.6%), and increase the pCR rate (27.7% vs. 13.8%) compared with the standard LCRT.

Recently, immune checkpoint inhibitors targeting the PD-1/PD-L1 pathway (PD-1/PD-L1 monoclonal antibodies) have provided a new treatment option for malignant tumours. PD-1/PD-L1 monoclonal antibodies can specifically bind to PD-1 or PD-L1 to block the PD-1/PD-L1 signalling pathway so that T cells can restore the immune response against tumours, thereby increasing the killing of tumour cells. PD-1/PD-L1 monoclonal antibodies have achieved significant efficacy in a variety of advanced cancers. MSI-H patients have mutations in mismatch repair genes and higher genetic instability, can produce more tumour neoantigens, and have a higher degree of T cell infiltration, leading to better immunotherapy efficacy [11, 12]. The Keynote-177 trial [13] has shown that for advanced CRC patients with microsatellite instability-high (MSI-H), PD-1 monoclonal antibody demonstrates excellent efficacy and safety. For early-stage CRC patients, clinical trials related to immunotherapy are also ongoing. The NICHE trial was the first clinical trial to perform immunotherapy (nivolumab and ipilimumab) in stage I-III colon cancers and showed a good response. Moreover, patients with MSI-H only account for 5%-20%, and the main CRC population is microsatellite-stable (MSS) patients. Improving the immunotherapeutic sensitivity of MSS patients remains a challenge.

Radiotherapy is one of the three major treatments for malignant tumours. Preclinical studies have shown that radiotherapy can induce the release of tumour neoantigens, promote the activation of antitumour T cells and the aggregation of tumour-infiltrating T cells, and cause the immunogenic cell death of tumour cells [14, 15]. Radiotherapy can induce the upregulation of PD-L1 expression in tumour tissues. The combination of radiotherapy with immunotherapy can relieve the immunosuppressive effect, enhance the secretion of T cell-derived antitumour cytokines, and enhance the efficacy of radiotherapy [16, 17]. Clinical studies of radiotherapy combined with immunotherapy also observed remote effects [18, 19], which are considered strong evidence that radiotherapy stimulates the body's antitumour immune response. Therefore, radiotherapy combined with immunotherapy is promising. Even in MSS CRC patients, radiotherapy is expected to increase their sensitivity to immunotherapy.

Based on the above evidence indicating the improved efficacy of the TNT strategy and the promising benefit of the combination of radiotherapy and immunotherapy, we are conducting a phase II trial of SCRT combined with CAPOX and Toripalimab for total neoadjuvant therapy in LARC patients. Patients will be treated with the TNT approach and divided into an inductive immunotherapy group and a consolidative immunotherapy group. We will explore whether SCRT combined with chemotherapy and Toripalimab can achieve better efficacy, good tolerance and prognosis in LARC patients.

### Methods/design

#### Study design

**Fig. 1:**
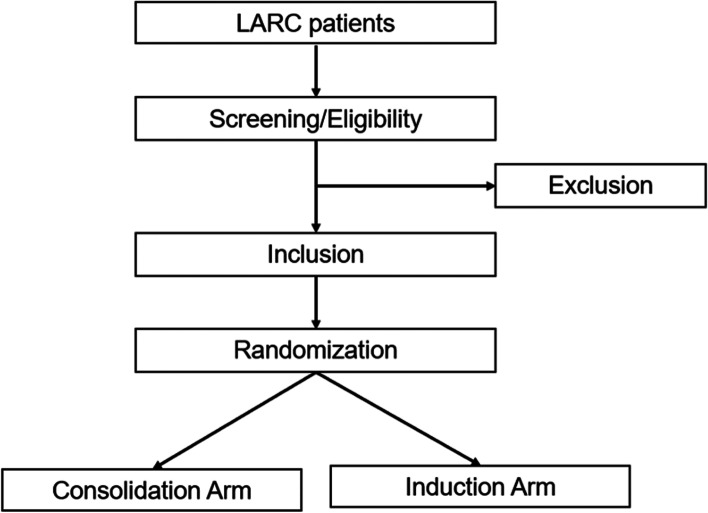
The patient recruitment and randomization process of TORCH study

The study is a multicentre, double-arm, prospective phase II trial of short-term radiotherapy combined with chemotherapy and immunotherapy in LARC. Patients will receive TNT regimens and be randomly assigned into two arms: a consolidation arm and an induction arm. Patients in the consolidation arm will receive short-term radiotherapy (25 Gy/Fx), followed by 6 cycles of capecitabine plus oxaliplatin (CAPOX) chemotherapy and Toripalimab, and finally receive TME surgery. Patients in the induction arm will first receive 2 cycles of CAPOX chemotherapy and Toripalimab, then receive short-term radiotherapy (25 Gy/Fx), followed by 4 cycles of CAPOX chemotherapy and Toripalimab, and finally receive TME surgery. The complete response (CR) rate (pCR rate plus cCR rate), adverse effects and long-term prognosis will be analysed. The study algorithm is presented in Fig. 1.

### Trial organization, ethics approval, drug supply and insurance

This trial is principal-investigator initiated by the Department of Radiation Oncology, Fudan University Shanghai Cancer Center. The study was approved by the Ethics Committee of Fudan University Shanghai Cancer Center (Approval Number: 2009224–7). A total of five other cancer centres in China are involved in the study, including Shanghai Changhai Hospital, Shanghai Changzheng Hospital, Shanghai East Hospital, Affiliated Hospital of Jiangnan University and People's Hospital of Tianjin. The study was also approved by the Ethics Committee of these hospitals. Toripalimab is provided free of charge by Shanghai Junshi Biomedical Technology Co., Ltd., which has purchased liability insurance for clinical trial subjects.

### Study population

LARC patients who meet the inclusion criteria and exclusion criteria (Table 1) are included in this clinical trial. Both MSS and MSI-H patients can be included in our study. However, the MSI status will be examined using colposcopy biopsy tissue to perform subgroup analyses retrospectively. The trial was opened on 13th April 2021, and the first patient was recruited on 6th May 2021.

**Table 1:** Inclusion and exclusion criteria

Inclusion criteria	Exclusion criteria
1. Pathological confirmed adenocarcinoma 2. Clinical stage T3-4 and/or N + 3. The distance from anal verge ≤ 12 cm 4. Without distance metastases 5. Age 18–70 years old, female and male 6. KPS > = 70 7. Baseline blood and biochemical indicators meet the following criteria: neutrophils ≥ 1.5 × 10^9/L, Hb ≥ 90 g/L, PLT ≥ 100 × 10^9/L, ALT/AST ≤ 2.5 ULN, Cr ≤ 1 ULN 8. With good compliance and signed the consent form	1. Pregnancy or breast-feeding women 2. Known history of other malignancies within 5 years 3. Known history of previous anti-tumor treatment, including radiotherapy, chemotherapy, immune checkpoint inhibitors, T cell-related therapy, etc 4. Known history of severe neurological or mental illness (such as schizophrenia, dementia or epilepsy) 5. Current severe cardiac disease (cardiac dysfunction and arrhythmia), renal dysfunction and liver dysfunction 6. Acute cardiac infarction or cerebral ischemic stroke occurred within 6 months before recruitment 7. Uncontrolled infection which needs systemic therapy 8. Active autoimmune disease or immunodeficiencies, known history of organ transplantation or systematic use of immunosuppressive agents 9. Known history of human immunodeficiency virus (HIV) infection (i.e., HIV 1 to 2 antibody positive), active syphilis infection, active pulmonary tuberculosis infection 10. Active Hepatitis B virus (HBV) or hepatitis C virus (HCV) infection at screening (i.e., HBsAg positive or HBV DNA positive, HCV RNA positive if anti-HCV antibody testing positive) 11. Allergic to any component of the therapy

### Treatment plan

**Fig. 2:**
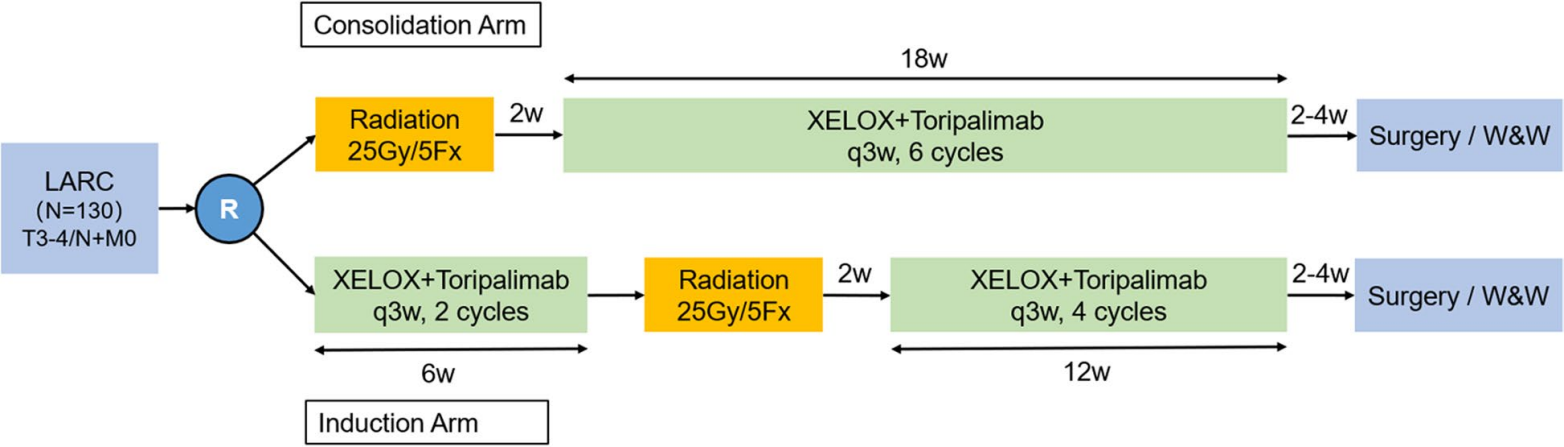
The two treatment regimens of TORCH study

Patients in the consolidation arm will first receive short-course radiotherapy. Two weeks after the completion of radiotherapy, six cycles of CAPOX and Toripalimab treatment will be performed. Patients in the induction arm will first receive two cycles of CAPOX and Toripalimab treatment and then receive short-term radiotherapy. Two weeks after the completion of radiotherapy, four cycles of CAPOX and Toripalimab treatment will be performed. The target volume is the rectal primary lesion and the pelvic lymphatic drainage area. The radiation dose is 25 Gy/5 Fx. The regimen of CAPOX and Toripalimab treatment includes oxaliplatin 130 mg/m^2^ d1, capecitabine 1000 mg/m^2^ bid d1-14, and Toripalimab 240 mg d1 (3 weeks per cycle). Surgery will be performed 2–4 weeks after the end of the whole neoadjuvant treatment. The specific surgical approach will be determined by the surgeon, including anterior resection (AR), abdominoperineal resection (APR), Hartman surgery or local excision. Adjuvant chemotherapy is not needed. If the patients achieve cCR after neoadjuvant therapy, a W&W strategy can be performed according to the decision of the patients. The treatment regimen is shown in Fig. 2.

### Study endpoints and assessment

The primary endpoint is the CR rate, that is, the rate of pCR plus cCR. The secondary endpoints include the grade 3–4 acute adverse effects rate, 3-year disease-free survival (DFS) rate, 3-year local recurrence-free survival (LRFS) rate, 3-year OS rate, rate of surgical complications and quality of life (QoL) scores.

The pCR status is defined as no viable tumour cells on the resected specimen. The specimen will be assessed by two independent pathologists. Pathological tumour regression grade (pTRG) will be evaluated according to the 8th American Joint Committee on Cancer (AJCC) Staging Manual. The pTRG and pCR status will be evaluated by two independent pathologists. If their conclusions are inconsistent, they will be evaluated again by a third pathologist. cCR is defined as undetectable tumour signs after nCRT by clinical examinations, including magnetic resonance imaging (MRI), endoscopy, and digital rectal exam (DRE).

Patients will receive regular examinations at the following time points: baseline, before the third immunotherapy, at the end of the whole neoadjuvant therapy (before surgery) and at every visit during the follow-up. The examinations include DRE, complete blood count, blood biochemical examination (aspartate aminotransferase, alanine aminotransferase, creatinine, blood urea nitrogen), thyroid hormone, adrenal hormone, myocardial enzymogram, serum tumour markers (CEA, CA199, AFP, CA724, CA242, CA50, etc.), and imaging examinations (pelvic MRI, abdominal CT and chest CT). Imaging efficacy will be evaluated based on the response evaluation criteria in solid tumours (RECIST v.1.1) and immune response evaluation criteria in solid tumours (iRECIST). cCR is defined as undetectable tumour signs after nCRT by clinical examinations, including MRI, endoscopy, and DRE.

Acute adverse effects will be classified at every cycle of treatment and recorded according to the Common Terminology Criteria for Adverse Events (CTCAE) 4.0. Quality of life will be evaluated at the end of the whole neoadjuvant therapy and at every two visits during the follow-up using the EORTC QLQ-C30 and EORTC QLQ-CR29 scales. At the same time, anorectal and bowel function will be evaluated using the Wexner score [20] and LARS score [21].

### Sample size

This study is a randomized, prospective, double-arm, phase II clinical trial. The primary endpoint of each group is the CR rate. The sample size was calculated using the pick-the-winner method, which was performed using R software (version 4.0.3; R Project for Statistical Computing, Vienna, Austria) with R package "power.ctepd". In this study, the reference CR rate is 25% (invalid response rate, p0), and we assumed that both treatment groups can achieve a CR rate of 40% (alternative response rate, p1). Patients will be randomly assigned to the two treatment groups at a ratio of 1:1. If a probability value of at least 95% needs to be reached to select the superior group, the sample size should be 106 patients (53 patients/group). Taking into account the maximum dropout rate of 20%, the final total sample size in this study will be 130 cases (65 cases per group).

### Follow-up

Patients will be scheduled for follow-up every 3 months after surgery until 3 years postoperatively. Physical examination, especially DRE, complete blood count, blood biochemical examination (aspartate aminotransferase, alanine aminotransferase, creatinine, blood urea nitrogen), thyroid hormone, adrenal hormone, myocardial enzymogram, and serum tumour marker (CEA, CA199, AFP, CA724, CA242, CA50, etc.) analyses, and imaging examinations (pelvic MRI, abdominopelvic CT and chest CT) will be performed every 3 months. Colonoscopy, late adverse effects collection, QoL assessment (EORTC QLQ-C30/CR29), Wexner score and LARS score will be performed every 6 months. The dates and sites of local recurrence and distant metastasis and the date and cause of death will be recorded in detail.

## Discussion

Currently, for LARC patients, a series of clinical trials of radiotherapy in combination with immunotherapy are ongoing. The main characteristics of these clinical trials include small sample size, phase II, single arm and single treatment sequence. However, their preliminary findings are promising. The Japanese VOLTAGE-A study [22] was the first reported trial. In this study, LCRT was used, followed by 5 cycles of nivolumab. The results showed that of 37 MSS patients, 11 (30%) reached pCR, 3 (8%) reached near pCR, and 1 patient reached cCR and finally undertook the W&W strategy. In addition, 3 of 5 MSI-H patients reached pCR (60%). The grade 3–4 immune-related toxicities were 7.7%. This result suggested that LCRT followed by immunotherapy could obtain a very good tumour response and good tolerance.

Another two trials investigated the efficacy of radiotherapy concurrent with immunotherapy. The ANAVA study [23] used 6 cycles of avelumab from the beginning of nCRT. Of the 96 patients who could be assessed pathologically, 22 (23%) reached pCR, 59 (61.5%) achieved pathological regression, and grade 3–4 immune-related toxicity rates were only 4%. The NRG-GI002 trial [24] adopted the TNT approach, in which the control group was 8 cycles of FOLFOX followed by LCRT (concurrent with capecitabine). The experimental group included 8 cycles of FOLFOX followed by LCRT (concurrent with capecitabine and Pembrolizumab). The pCR rates were 29.4% and 31.9% (P = 0.75), and the cCR rates were 13.6% and 13.9% (P = 0.95). Although the tumour regression rates were similar, the pCR + cCR rates of both groups were as high as 44%; that is, nearly half of the patients achieved complete regression, indicating the promising efficacy of TNT. However, the addition of immunotherapy failed to further improve the tumour regression grade, which is worthy of further consideration. The lymphocytes are sensitive to radiation. When LCRT is combined with concurrent immunotherapy, radiation may kill locally aggregated or activated lymphocytes, thus adversely affecting the immune response. Thus, sequential combination of radiotherapy and immunotherapy may be better than concurrent combination of them.

In terms of SCRT, there are fewer clinical trials. The Averectal trial [25] performed SCRT followed by 6 cycles of mFOLFOX6 and avelumab and found that 37.5% of the patients achieved pCR and 30% of patients achieved near-pCR (TRG 1). The major pathological response rate was 67.5%. No grade 3–4 immune-related adverse effects were observed. Another Chinese study that used SCRT followed by 2 cycles of XELOX and camrelizumab published their findings recently [26]. Among the 27 patients (26 MSS and 1 MSI-H), the pCR rate was as high as 48% (46% in the MSS group and 100% in the MSI-H group). The rates of R0 resection, anal preservation and stage reduction were 100%, 89% and 70%, respectively. No grade 3–4 immune-related adverse effects were observed, and the grade 3 haematological toxicity was alleviated after treatment. Furthermore, a phase III clinical trial (NCT04928807) is now open at this cancer centre, comparing the efficacy of SCRT and sequential camrelizumab and chemotherapy with LCRT and sequential chemotherapy.

The above evidence showed very promising pCR rates and good tolerance for the combination of radiotherapy and immunotherapy, both LCRT and SCRT. To date, SCRT combined with immunotherapy seems to have superior pCR rates compared with LCRT. An ongoing phase II clinical trial (PRIME-RT) is treating patients with the TNT approach and comparing the disparities of LCRT and SCRT, which will provide us with more information about the two treatment models. PRIME-RT allows cCR patients after nCRT to receive the W&W strategy, which is similar to our regimen. Compared with conventional fractionation radiotherapy, several studies have shown some advantages of hypofractionated radiotherapy. It was reported that hypofractionated radiotherapy can inhibit the recruitment of myeloid-derived suppressor cells (MDSCs) to tumours and achieve better tumour growth inhibition than conventional fractionation in mice [27]. The combination of hypofractionated radiotherapy and immunotherapy can induce remote effects in mice [28]. Moreover, hypofractionated radiotherapy has little effect on peripheral blood lymphocytes [29] and has fewer acute radiotherapy-related adverse effects. The above findings provide a theoretical basis for our design of SCRT in combination with immunotherapy.

As previously mentioned, multiple clinical trials have demonstrated that the TNT approach has many advantages in terms of tumour regression after nCRT and long-term prognosis. Thus, we would like to combine the TNT strategy and immunotherapy in LARC patients. Unlike the NGI-002 trial, we adopted the sequential combination model of SCRT and immunotherapy to investigate whether the addition of immunotherapy to the TNT approach could lead to improved tumour regression and prognosis.

Furthermore, the better sequence of radiotherapy and immunotherapy, that is, whether induction therapy or consolidation therapy is better, is undetermined and is worth exploring. The OPRA trial [9] showed that the consolidation arm achieved a higher organ preservation rate than the induction arm (58% vs. 43%). The CAO/ARO/AIO-12 trial [30] also compared the induction arm and consolidation arm. It performed 3 cycles of FOLFOX before or after long-course nCRT and finally found that compared with the induction arm, the consolidation arm achieved a higher pCR rate (25% vs. 17%), a lower rate of grade 3–4 toxicities (27% vs. 37%) and better compliance with neoadjuvant treatment. We believe that the use of radiation before immunotherapy can produce more tumour neoantigens to promote the effects of subsequent immunotherapy. The use of immunotherapy can change the microenvironment of tumours to promote the effects of radiotherapy. In most current clinical trials, the PD-1 antibody is usually used after or during radiotherapy (consolidative or concurrent immunotherapy), and nearly no induced immunotherapy is used. To date, TORCH is the first clinical trial to use the TNT strategy and compare the efficacy, adverse response and prognosis of induced immunotherapy and consolidative immunotherapy. We will use the “pick the winner” method to select the strategy with a better CR rate.

For the present trial, the primary endpoint is the CR rate (the rate of pCR plus cCR). Based on the average CR rate (25%) achieved by previous nCRT regimens, we assume that both treatment groups can achieve a CR rate of 40%, according to the primary results of recent trials. The other important issues that we care about are adverse effects, especially grade 3–4 neutropenia, thrombocytopenia, hepatic dysfunction and diarrhoea. Although we chose the short course, which has fewer acute toxicities, the overlapping effects of radiation, chemotherapy and immunotherapy still deserve attention. It has been reported that immune-related colitis will occur at 5–10 weeks of immunotherapy, so the abdominal symptoms of patients during this period should be monitored, especially for the induction arm, because radiotherapy is used at the 7th week. In addition, given that oxaliplatin often results in thrombocytopenia, it is possible to cause grade 3–4 thrombocytopenia after 3 cycles of CAPOX and Toripalimab, which should be considered. The rates of adverse effects occurring in the abovementioned clinical trials were relatively low, especially immune-related adverse effects, which gave us great confidence.

In summary, TORCH is a randomized, prospective, multicentre, double-arm, phase II trial of short-course radiotherapy combined with CAPOX and Toripalimab for total neoadjuvant therapy in LARC patients to investigate whether the addition of immunotherapy to nCRT can bring improved tumour regression, good tolerance and better prognosis. In this trial, the TNT strategy is adopted, and the induction arm and consolidation arm are compared. We look forward to obtaining improved results and selecting a better treatment strategy.

## Data Availability

Not applicable in this study protocol. The data (such as efficacy and toxicity) produced during and after the trial are available from the corresponding author upon reasonable request.

## Abbreviations

AJCC: American Joint Committee on Cancer
CAPOX: Capecitabine plus oxaliplatin
Ccr: Clinical complete response
CR: Clinical response
DRE: Digital rectal examination
LARC: Locally advanced rectal cancer
LCRT: Long-course radiotherapy
MDSC: Myeloid-derived suppressor cells
MRI: Magnetic resonance imaging
MSI-H: Microsatellite instability-high
MSS: Microsatellite-stable
NCCN: National Comprehensive Cancer Network
Ncrt: Neoadjuvant chemoradiotherapy
OS: Overall survival
pCR: Pathological complete response
RECIST: Response evaluation criteria in solid tumours
SCRT: Short-course radiotherapy
TNT: Total neoadjuvant therapy
TRG: Tumour regression grade
W&W: Watch and Wait
